# Inflammatory Cardiovascular Risk Biomarkers: Update on Novelties and Limitations

**DOI:** 10.1155/2012/515692

**Published:** 2012-06-04

**Authors:** Fabrizio Montecucco, François Mach, Aldo Pende, Thomas H. Schindler, Rafaela F. da Silva, Nicolas Vuilleumier

**Affiliations:** ^1^Division of Cardiology, Department of Medical Specialties, Geneva University Hospitals and University of Geneva, Avenue de la Roseraie 64, 1211 Geneva, Switzerland; ^2^First Medical Clinic, Laboratory of Phagocyte Physiopathology and Inflammation, Department of Internal Medicine, University of Genoa, Viale Benedetto XV 6, 16100 Genoa, Italy; ^3^Laboratory of Hypertension, Department of Physiology and Biophysics, Federal University of Minas Gerais, Belo Horizonte 31270-901, Brazil; ^4^Division of Laboratory Medicine, Department of Genetics and Laboratory Medicine, Geneva University Hospitals, Rue Gabrielle-Perret-Gentil, 1211 Genera, Switzerland

Evidence from Framingham studies showed that some disorders and conditions (such as hypertension, hyperlipidaemia, smoking, diabetes, old age, and male sex) are particularly useful to estimate the cardiovascular (CV) risk of acute ischemic events [1] and are currently considered as the “traditional” cardiovascular risk factors. This led to the development of several clinically based CV risk stratification tools, and the Framingham risk score is one of the most commonly used CV risk stratification tools nowadays [2]. However, these “traditional” cardiovascular risk factors were shown to be suboptimal for proper CV risk stratification due to low specificity and sensitivity [3–6]. Therefore, a novel concept of “global” cardiovascular vulnerability has been suggested to better predict acute cardiovascular events [7, 8]. Interestingly, this approach was particularly focused on “the clinical and laboratory complexity of the patient” instead of “a single risk factor.” Several pathophysiological parameters have been proposed as new cardiovascular risk factors potentially improving the assessment of patient vulnerability [7, 8]. Although some controversies still exist, the atherosclerotic role of inflammatory biomarkers (such as C-reactive protein (CRP), cytokines, and chemokines) [9–12] has been shown within atherosclerotic plaques, in the systemic circulation or in the peripheral ischemic tissues in both *in vivo* and *in vitro *models. More recently, novel inflammatory mediators (such as circulating autoantibodies and hormones) have been also identified [13, 14]. These soluble mediators have been shown to trigger several atherosclerotic functions of both inflammatory and vascular cells [15]. On the other hand, the mobilization of protective cell subsets might also counterbalance atheroprogression, thus limiting the chronic inflammatory processes and improving cardiovascular outcomes [16]. These protective aspects might be particularly relevant when atherosclerosis is associated with concomitant inflammatory conditions (such as rheumatoid arthritis, infections, and diabetes), which seem to further accelerate atherogenesis towards final acute ischemic complications or arterial aneurysms [17–19]. This special issue focused on new soluble mediators as promising candidates to better assess the cardiovascular risk. Importantly, the limitations of the potential clinical use of these systemic and intraplaque inflammatory molecules influencing atheroprogression have also been discussed. In particular, E. Lupia and coworkers revised the clinical role of thrombopoietin (a humoral growth factor activating platelets) as a promising biomarker of cardiovascular injury. P. Kunes and colleagues developed interesting findings on the controversial role of pentraxin family (which includes CRP) in the inflammatory response. The authors focused on the newly discovered pentraxin 3 and suggested paradoxical issues that will probably be validated in the near future. Drs. D. Vasic and the D. Walcher from the University of Ulm (Germany) revised the potential proatherosclerotic activity of C-peptide as a predictor of cardiovascular risk in diabetic subjects. This paper focused on a hot-topic issue in cardiovascular research. In fact, only very recently, C-peptide (previously considered as a product of cleavage of proinsulin) has been proposed as an active factor favouring atherosclerosis. Despite some limitations on the molecular mechanisms (the C-peptide receptor remains to be identified), this molecule might activate different leukocyte subsets in atherogenesis. Among these cells, the different types of macrophages appear as a relevant target for inflammatory mediators. T. Gui and coworkers discussed this issue and provided an interesting and comprehensive review on the impact of macrophages in both early and advanced phases of atherogenesis. The authors also suggested these cells as promising biomarkers of plaque vulnerability. Another review article of the present issue mainly focused on the proteolytic mechanisms regulating intraplaque remodelling potentially favouring the formation of abdominal aortic aneurysms. Drs. Z.- Z. Li and Q.-Y. Dai focused on direct activities mediated by nicotine via its receptor (nicotinic acetylcholine receptor) on plaque inflammation, angiogenesis, and smooth muscle cell dysfunction. The paper of Z. Qu and colleagues further developed the proinflammatory reactions underlying abdominal aortic aneurysm formation in advanced atherosclerosis. Some receptors of sphingosine-1-phosphate (S1P, a recently discovered lysophospholipid) were shown to play a crucial role in human abdominal aorta aneurysms as compared with normal aorta control tissues. In particular, S1P3 receptor was significantly upregulated in human abdominal aortic aneurysms, while S1P2 receptor was downregulated as compared to normal aortic samples. Although the molecular mechanisms remain unexplored (parallel expression of other inflammatory mediators was not investigated), this observational study identified a potential novel cardiovascular biomarker (S1P) in advanced atherosclerosis. Differently from this paper, K.-Karatolios and coworkers focused their study on well-known cytokines and growth factors. Surprisingly, the authors showed that the levels of vascular endothelial growth factor (VEGF) and human basic fibroblast growth factor (bFGF) in pericardial effusions of patients with autoreactive or viral inflammation were significantly higher as compared to patients with coronary artery disease (CAD). No significant difference was shown for inflammatory cytokines. Although the underlying molecular mechanisms remain to be investigated, these two growth factors might be more promising biomarkers of pericardial inflammation than “traditional” cytokines. After a diffuse discussion on soluble mediators potentially increasing the cardiovascular risk, R. Wyderka and coworkers focused their investigation on the mobilization of protective CD34+CXC4+ stem/progenitor cells in humans after an acute myocardial infarction. The authors clearly showed that this process was positively correlated with the improvements of values of the left ventricular ejection fraction at 1-year of followup, suggesting a beneficial activity of these cells in myocardial repair.

In both inflammatory micro- and macroenvironments characterizing atherosclerosis, some tissues might also produce some unexpected molecules potentially contrasting with physiological paradigms. M. L. Sirico and coworkers showed that human adipocytes can express and synthesize albumin. This paper was selected to highlight the potentialities of adipose tissue as an inflammatory organ capable of ectopically producing a large variety of mediators during atherogenesis. The present issue includes by the paper of C. Falcone and colleagues investigating the potential activity as biomarkers of soluble Receptor for Advanced Glycation End products (sRAGE) in patients with hypertension and increased cardiovascular risk. The authors showed that antihypertensive treatments might affect sRAGE plasma levels. All the papers included in the present issue focused on both novelty and limitations of promising inflammatory biomarkers that in the near future might be used in the clinical practice to improve cardiovascular disease prevention. We hope that the reader will find some useful inputs for developing research and updating knowledge on cardiovascular pathophysiology.
